# Optimized Mass Spectrometry Analysis Workflow with Polarimetric Guidance for *ex vivo* and *in situ* Sampling of Biological Tissues

**DOI:** 10.1038/s41598-017-00272-y

**Published:** 2017-03-28

**Authors:** Michael Woolman, Adam Gribble, Emma Bluemke, Jing Zou, Manuela Ventura, Nicholas Bernards, Megan Wu, Howard J. Ginsberg, Sunit Das, Alex Vitkin, Arash Zarrine-Afsar

**Affiliations:** 1 Techna Institute for the Advancement of Technology for Health, University Health Network, Toronto, ON M5G-1P5 Canada; 2 Department of Medical Biophysics, University of Toronto, 101 College Street Suite 15-701, Toronto, ON M5G 1L7 Canada; 3 Peter Gilgan Centre for Research and Learning, Hospital for Sick Children, 686 Bay Street, Toronto, ON M5G-0A4 Canada; 4 Department of Surgery, University of Toronto, 149 College Street, Toronto, ON M5T-1P5 Canada; 5 Keenan Research Center for Biomedical Science, Li Ka Shing Knowledge Institute, St. Michael’s Hospital, 30 Bond Street, Toronto, ON M5B-1W8 Canada; 6 Institute of Biomaterials and Biomedical Engineering, University of Toronto, 164 College Street, Toronto, ON M5S 3G9 Canada; 7 Department of Radiation Oncology, University of Toronto, 610 University Avenue, Toronto, Ontario M5G 2M9 Canada; 8 Division of Biophysics and Bioimaging, Ontario Cancer Institute, University Health Network, 610 University Ave, Toronto, ON M5G 2M9 Canada

## Abstract

Spatially Targeted Mass Spectrometry (MS) analysis using survey scans with an imaging modality often requires consecutive tissue slices, because of the tissue damage during survey scan or due to incompatible sample preparation requirements between the survey modality and MS. We report two spatially targeted MS analysis workflows based on polarized light imaging guidance that use the *same* tissue sample for survey and targeted analysis. The first workflow is applicable for thin-slice analysis, and uses transmission-polarimetry-guided Desorption ElectroSpray Ionization Mass Spectrometry (DESI-MS), and confirmatory H&E histopathology analysis on the same slice; this is validated using quantitative digital pathology methods. The second workflow explores a polarimetry-guided MS platform for thick tissue assessment by developing reflection-mode polarimetric imaging coupled with a hand-held Picosecond InfraRed Laser (PIRL) MS ablation probe that requires minimal tissue removal to produce detectable signal. Tissue differentiation within 5–10 s of sampling with the hand-held probe is shown using multivariate statistical methods of the MS profiles. Both workflows were tasked with differentiating necrotic cancer sites from viable cancers using a breast tumour model, and their performance was evaluated. The use of the same tissue surface addresses mismatches in guidance due to intrinsic changes in tissue morphology over consecutive sections.

## Introduction

Mass spectrometry (MS) is a promising technology for detailed characterization of the molecular composition of biological tissues, with low detection limits resulting in high sensitivity and specificity. MS can both ‘image’ thin slices of *ex vivo* tissue and perform ‘point sampling’ of thick tissue *in situ* ^1, 2^.

Ambient MS techniques such as Rapid Evaporative Ionization Mass Spectrometry (REIMS), Laser Ablation Electrospray Ionization (LAESI) and Desorption Electrospray Ionization (DESI) Mass Spectrometry have now reached widespread utility in characterization of biological samples. Applications of ambient MS include cancer site imaging from slices of *ex vivo* tissue^1, 3–5^, or cancer profiling for the identification of cancer type or subtype^1, 2, 6–16^. For these applications, known or determined cancer MS profiles (i.e., *m/z* values unique to cancer type or subtype) form the needed contrast, and the MS probe should be directed to the disease site (or margins) to speed up the procedure and minimize the time analyzing otherwise healthy tissues. However, the majority of MS imaging and profiling approaches are spatially untargeted, and numerous measurements may be required to correctly identify the cancer subregion for subsequent detailed characterization, resulting in long examination times^17^. An approach for targeted collection of MS data from disease sites or other regions of interest would reduce analysis time, and will enable this powerful technology to fulfill its clinical potential for *rapid*, sensitive and accurate disease diagnosis. Current advances in MS analysis speeds, through improvements in hardware and software platforms that control raster scanning, may also prove useful in this regard.

We have recently identified polarized light imaging (polarimetry) as a suitable targeting technology for MS, potentially capable of a significant time reduction for MS imaging and analysis of biological tissues^18^. For efficient guidance of DESI-MS imaging using thin tissue slices, it is desirable to use the same slice for polarimetry guidance, MS analysis and subsequent histopathology staining; the last step is necessary for ground-truth confirmatory imaging^3, 5, 6, 19–22^. Our initial research on polarimetry-guided MS made use of multiple tissue sections^18^, adding complexity to the workflow, wasting tissue, and contributing to potential validation inaccuracies due to both tissue heterogeneity along the sectioning axis^7^ and sample preparation artifacts. Further, the proof-of-principle study^18^ did not explore the *in situ* thick tissue scenario. It is important to note that other wide-field imaging techniques such as Raman spectroscopy^23^, fluorescence imaging and optical coherence tomography (OCT) can also be used as the survey imaging modality to guide the acquisition of targeted MS images.

We hereby present two optimized, polarimetry-guided MS workflows that allow efficient analysis of *ex vivo* and *in situ* thick tissue. The first addresses the thin-slice-scenario limitations of the previous study^18^, providing an optimized platform for ‘transmission mode’ polarimetry-guided MS imaging with pathology confirmation in a single tissue slice. The utility of this optimized methodology is demonstrated by differentiating necrotic from viable tumour tissue in breast cancer xenograft models subjected to DESI-MS^21^. This MS method, where charged microdroplets of solvent incident on a thin tissue slice desorb and ionize small molecule lipids and metabolites, creates a unique MS profile for cancer typing and tumour subclass identification in a variety of cancers^1, 5, 6, 19, 24, 25^. Here we use a newly engineered DESI spray outlet^26, 27^ that allows efficient and minimally destructive desorption ionization from thin tissue slices (e.g., 10 µm). The minimally destructive nature and reduced tissue thickness requirements allow subsequent histologic staining on the same MS-imaged tissue slice for detailed microscopy evaluation, provided suitable solvent compatible with post DESI-MS staining protocols is used^28^. Coupled with optimized transmission-mode polarimetry imaging for MS guidance, we are thus now able to perform all three examinations on the same slice. These methodological developments are important for clinical translation of polarimetry-guided MS for rapid and accurate pathology assessment in thin sections of excised tissues.

The second workflow moves beyond thin tissue sections and explores the use of ‘reflection mode’ polarimetry combined with a novel, hand held MS point sampling probe utilizing Picosecond InfraRed Laser (PIRL)^29^ ablation for tissue profiling with MS through guided point sampling. This workflow is used to examine the surface of thick tissue *ex viv*o, and extends the pioneering works of the Takats group in rapid tissue profiling with surgical aerosols produced with diathermy, lasers and ultrasonic aspiration methods^2, 12–14^. Unlike electrocautery approaches that produce aerosolized tissue material for real time capture and MS analysis^2, 9, 16^ PIRL uses a “cold” ablation laser that does not thermally damage tissue surrounding the sampling site, with minimal amounts of post ablation scar tissue and avoidance of the cellular stress response^30^. We thus anticipate that a cold ablation scalpel may have utility in negative cancer margin assessment or tumour bed examinations where the damage to the healthy tissue due to sampling must be kept to a minimum. The polarimetry-guided PIRL-MS examination thus does not substantially alter the examined tissue, an important practical consideration for clinical translation to thick tissues *in situ* and potentially *in vivo*. PIRL has been previously used by our group in biological tissue imaging after coupling to post ablation Electrospray ionization (PIR-LAESI)^31^. Laser Ablation Electrospray Ionization (LAESI) using nanosecond mid infrared laser radiation is an emerging ambient ion source being investigated for biological tissue imaging^32^.

The reported developments should prove useful for advancing MS as a rapid and accurate molecular pathology tool complementary to current tissue examination methods, in a variety of preclinical and future clinical scenarios. The proposed workflows were validated using experimental polarimetry and MS analysis with pathology verification using a xenograft breast cancer model containing necrotic and viable cancer sites. The same slice of this tumour was subjected to DESI-MS analysis, polarimetry in the transmission mode and histological staining. The concordance of signal from all three modalities was examined. Polarimetric imaging in the reflection mode, compatible with thick tissue work, was also performed. Online integration between PIRL ablation and MS was completed, and polarimetric measurements in the reflection mode were sufficient to guide the PIRL probe to areas of necrosis in anticipation of future work with bulk tissues. The utility of MS profiling with the hand held PIRL-MS probe for rapid differentiation of tissue types was demonstrated using mouse organs through multivariate statistical analysis methods.

## Experimental Methods

### Animal models and sample preparation

All animal studies were conducted in accordance with institutional guidelines and approved by the animal use committee (Animal Use Protocol (AUP) at the University Health Network, Toronto). This study repurposes some of the samples developed and analyzed in a previous work from our laboratory^33^. Some of the MS or histology images originally reported in that work have been reproduced here for the clarity of the discussion.

The LM2-4 human breast cancer tumour model was established in female Severe Combined ImmunoDeficient (SCID) mice (Harlan). The mice were inoculated in their left inguinal mammary fat pad with 4–5 × 10^6^ cells. The animals were then housed for 2–3 weeks to allow the primary tumour to reach a volume >250 mm^3^ (measured by calipers). Primary tumours were surgically removed, and mice were housed for a few weeks to allow metastasis. Metastatic tumours that appeared in the axillary lymph nodes in the upper limb were surgically excised, flash frozen over liquid N_2_ vapour and stored at −80 °C for subsequent cryosectioning.

Tumours were mounted onto a metal specimen holder of cryostat with a small amount of Tissue-Tek, glycol based Optimal Cutting Temperature (OCT) compound (Sakura Finetek) to provide support. Slices 10 and 50 µm thick were prepared using a CM1950 cryostat (Leica), and mounted onto Superfrost Plus microscope slides. The slides were stored at −80 °C until analyzed.

*In vivo* mouse tissue profiling study used NOD SCID gamma (NSG) mice (Jackson Laboratory). Mice were maintained in accordance with Toronto Centre for Phenogenomics (TCP) institutional animal protocols, and sacrificed by CO_2_ inhalation. Organ tissues were dissected, and kept on ice for further analysis. Animal Use Protocol (AUP) was approved by the TCP committee under AUP 0293 H. Tissue water content values (in rats) can be found in this reference^34^.

### DESI-MS Imaging

In this study, we performed DESI-MS using a Xevo G2XS Quadrupole-Time-Of-Flight Mass Spectrometer (Q-TOF-MS from Waters) in the negative ion mode using the ‘sensitivity’ setting. A commercial DESI-MS source from Waters was used with no modifications. The microscope slides containing tissue sections were mounted on a 2D slide scanning stage and subjected to DESI-MS imaging in the negative ion mode. A mass range of *m/z* 200 to 1000 was used. The spray solvent used was a 1:1 mixture of acetonitrile and dimethylformamide (HPLC-MS grade, Sigma Aldrich) with Leucine Enkephalin (150 pg/μL). The spray solvent was delivered at a flow rate of 1 μL/min. The source settings are as described previously^35^. The stage moved at a constant velocity of 100 μm/s. While a combination of increased scan rate and potentially more efficient solvent systems that allow extraction in a short time without loss of sensitivity can be explored to provide faster acquisition of DESI-MS spectra, all images reported in this work were acquired using the above mentioned parameters. MS spectra were integrated for 1 s, resulting in a spatial resolution of 100 μm for our images. Spectra were calibrated for mass accuracy using the mass of Leucine Enkephalin in the solvent spray. DESI-MS ion images (normalized by Total Ion Current) were created using High Definition Imaging (HDI) package from Waters (500 most abundant peaks). The software allowed extraction of spectra from user defined ROIs. The spectral data from ROIs on the images was exported to MassLynx (Waters) for display and lock mass correction.

### PIRL ablation MS

A 2 m long Tygon tube with an inner diameter of 1.6 mm (McMaster Carr) was attached to the collection capillary of a commercial DESI-MS interface (Waters). The length (2 m) was sufficient to reach the analysis table without blocking instrument access. Diffusion of heat from the ion block proved sufficient to facilitate desolvation of phospholipids and fatty acids extracted from the tissue with PIRL. The capillary can also be heated at the bend to improve sample desolvation. Laser ablation was performed at a wavelength of 3,000 ± 100 nm with ~250 mW of power from the tip of a 2 m long flexible multimode sapphire fiber with core diameter of 425 μm that was coupled to a commercial solid state picosecond mid IR laser (Model PIRL 3000, Attodyne Lasers). The laser was operating at 1 kHz with pulse duration of 300 ± 100 ps. The laser tip was manually rastered across the tissue surface with a typical speed of ~2–10 mm/s and a tip to surface distance of ~1 mm. The ablation plume was collected by holding the collection tube 1–2 mm from the ablation surface. The fluence (average power/spot size) was calculated based on measured output of the laser at the tip, and the laser spot of ~500 μm (approximately collimated beam after the fiber). Since the laser beam was fairly collimated to within 5 mm after the tip, this operation geometry produced an ablation fluence of ~0.15 J/cm^2^. Typically, reasonable MS spectra with good signal to noise ratios were obtained from interrogating a ~1–5 mm^2^ area with 5–10 s of sampling. MS analysis was performed in the negative ion mode. Because of manual movement and varying speed, we are unable to provide typical ablation depth information. However, estimating from typical speed of movement we anticipate a depth of 300 μm. Characterization of open beam PIRL laser ablation using a controlled imaging setup with translation stages is presented in^29^.

### Statistical Analysis

MS peak lists (from *m/z* 200 to *m/z* 1000) were uploaded into the Metaboanalyst 3.0 web portal, with a mass tolerance of 20 ppm. Features that contained greater than 80% missing values were removed, and the remaining missing values were estimated using PPCA. The data were then filtered by Interquantile range (IQR)^36^. The ion abundances were normalized to the sum of *m/z* intensities for each spectrum, and then subjected to Pareto scaling^36^. Partial Least Squares Discriminant Analysis (PLS-DA) was performed to examine the grouping of MS profiles for different mouse tissue types^37, 38^.

### Polarimetry

Polarimetry imaging was performed at 635 nm using a homemade wide-field polarimetry system based on rotatable polarizers, removable quarter wave-plates, and CCD camera, as previously described^18^. The system was calibrated using the eigenvalue calibration method^39^. Images were taken in both transmission, and reflection mode (~25 degrees off exact backscattering) geometries. For each sample, a set of 24 polarization-resolved images were taken under different combinations of input and output polarization states. This set of polarization images then used to calculate the sample Mueller matrices (polarimetry transfer functions) at each pixel using standard linear algebra inversion techniques. From the sample Mueller matrices, the polarization metric of depolarization was determined using Lu-Chipman Mueller matrix decomposition^40^. Acquisition of each set of polarization images took less than 5 minutes. The field-of-view (FOV) was approximately 1.7 cm × 1.7 cm, with a pixel size of approximately 17 μm × 17 μm.

### Digital Pathology and Segmentation of DESI-MS images

Digital Hematoxylin and Eosin (H&E) pathology images in the form of pyramid files containing 20x magnification images of the H&E stained slides (pixel resolution of 0.5 microns/pixel), were loaded into Tissue Studio (Definiens AG, Munich, Germany). A machine-learning algorithm built in the software package was used to separate and classify viable and necrotic tumour regions-of-interest (ROIs) from surrounding healthy muscle tissue. This algorithm developed a classifier solution using 5–10 tissue segments manually assigned by a pathology trained user as necrotic or viable etc, and applied the solution to the entire area of tissue sections without further intervention. These classifications used a deconvolution of hematoxylin and Eosin stain signals. The nuclear recognition was based on thresholding of the hematoxylin signal. Total areas of each ROI and their spatial distributions were calculated as percent total area of the segmented image.

## Results and Discussion

**Figure 1:**
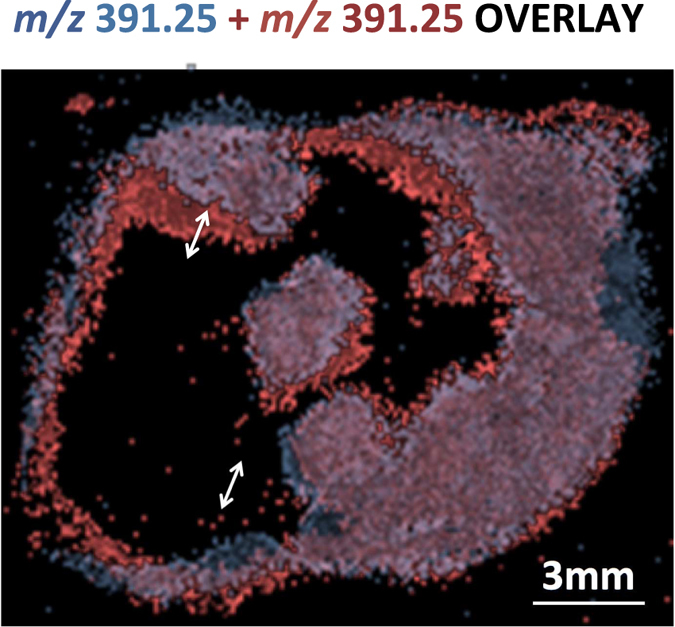
Changes in tissue morphology along the axis of tissue sections. Overlay of the distributions of viable cancer marker ion (*m/z* 391.25 from DESI-MS imaging) from two serial sections of human breast cancer tumour ~75 μm apart. Images were overlaid using optimized rigid body alignment, as judged by largest circumference of aligned regions on outside border of the tissue. The analysis reveals a significant (1–1.5 mm) mismatch of internal borders between consecutive viable cancer subregions. The DESI-MS image of the marker shown is published previously^33^ and is reproduced here for the clarity of our discussion regarding how tissue morphology in this tumour model changed over the 75 μm axial distance.

Our initial proof-of-principle polarimetry-guided MS workflow used a 50 μm tissue slice for optimal polarimetry imaging, a 20 μm tissue slice for DESI-MS imaging, and a consecutive 5 μm slice in the middle for pathology assessment^18^. Thus the workflow for best quality imaging used signals from a combined ~75 μm thick tissue section subjected to three imaging modalities of polarimetry, DESI-MS and H&E staining for validation. In addition to wasting tissue and the inconvenience of multiple sections, this methodology assumes that tissue microstructure stays constant over this spatial scale. Figure 1 illustrates the potential risks associated with this assumption across the previously used length scale of 75 μm^18^. By overlaying DESI-MS images of *m/z* 391.25 (a biomarker ion for viable breast cancer cells^33^) distributions, from serial sections 75 μm apart, we illustrate how morphology and composition of viable breast cancer cell regions^33^ vary with depth along the sectioning axis. As seen, tissue spatial heterogeneity results in mis-alignments on the order of 1.0–1.5 mm in some regions. This may be significant, and for example exceeds the tolerance level in resection margins for routine breast cancer procedures^41, 42^. Therefore, the previous implementation of our approach^18^ could lead to false positives (potentially resulting in unnecessary resection of healthy tissue) or false negatives (potentially resulting in cancer recurrence due to incomplete removal of viable cancer cells). The use of the *same* tissue slice for polarimetric guidance, MS interpretation, and correlation with pathology would be greatly beneficial for improved methodology performance, tissue conservation, and convenience.

Polarimetry-guided DESI-MS imaging (with histology validation) of a single tissue slice requires various technological improvements, including: (1) optimization of DESI-MS sprayer^26, 27^ to provide gentle desorption which avoids tissue damage, while still providing sensitive detection with sufficient signal from the thinner tissue sections; (2) optimization of DESI-MS solvent conditions^28^ to be compatible with post DESI-MS histology staining; (3) increased sensitivity of polarimetry detection to enable sufficient contrast using histology-compatible thin tissue sections; and (4) added functionality to the DESI-MS acquisition interface to allow co-registration of image coordinates for targeted collection of DESI-MS data. This work brings together these various improvements to enable improved targeted analysis of tissues using the polarimetry – DESI-MS tandem.

**Figure 2:**
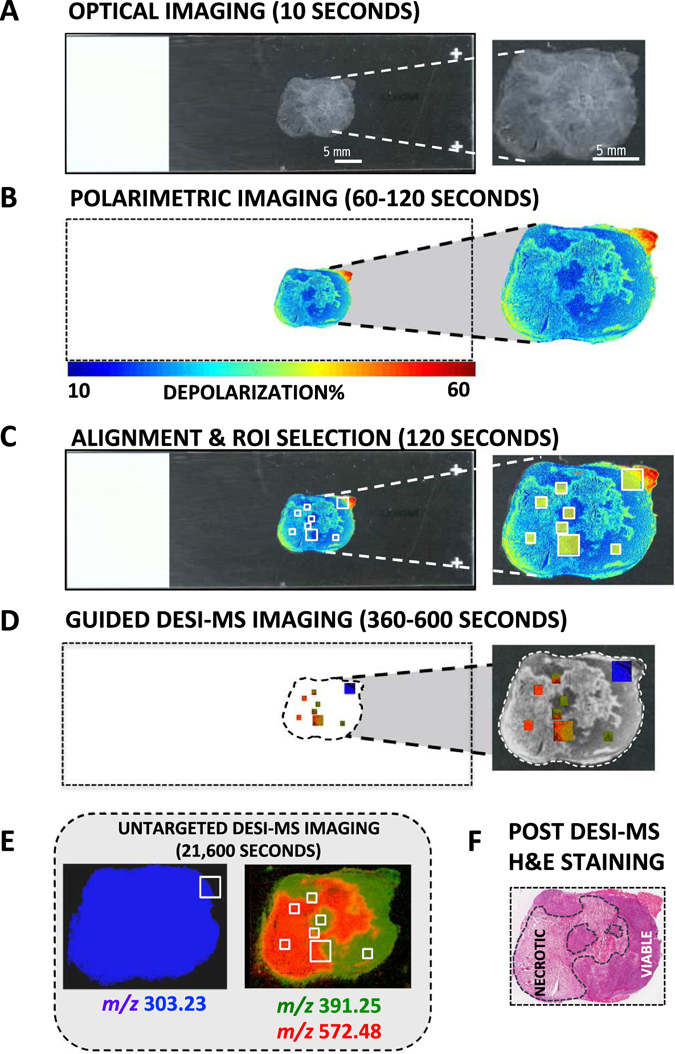
Visualization of the steps in optimized polarimetry guided DESI-MS workflow. (**A**) Optical image of tissue section with microscope slide corners included. (**B**) Wide-field polarimetry image of the tissue slice, identifying regions of different depolarization. (**C**) Polarimetric image is aligned with optical image and regions of interest (ROIs), 1 and 4 mm^2^ in size, identified by polarimetry are selected for DESI-MS analysis. (**D**) DESI-MS imaging of the targeted ROIs is performed. (**E**) Untargeted DESI-MS image of the entire slice (after targeted imaging). The markers displayed in D, E represent viable cancer (green, *m/z* 391.25), and necrotic cancer (red, *m/z* 572.48). The marker *m/z* 303.23 (blue) is present in many tissue types but is much more common in cancer (necrotic and viable) than in muscle. Even with the more conservative estimates, the guided workflow is more than 20 times faster than untargeted imaging of the whole sample. (**F**) Image of the same slice stained by H&E, after DESI-MS analysis, for validation of results.

Figure 2 summarizes the resultant optimized workflow using a single 10 µm tissue section. Relevant experimental and analysis times are also indicated. The examined tissue was cancer infiltrated lymph node derived from a murine xenograft tumour model of human breast cancer, containing viable and necrotic cancer regions. The tissue slice was mounted on a microscope glass slide and optically imaged such that the borders of the slide were visible. These borders were used in conjunction with the subsequent polarimetry image to provide a coordinate system for DESI-MS, using rigid body methods^43^. The slide was then subjected to wide-field Mueller matrix polarimetry imaging^44^. One or more Mueller matrix parameter images, such as tissue depolarization^44^, are used to identify regions of suspected pathology or regions of heterogeneity present in the tissue. The composite (optical and polarimetry overlay) image was loaded into the DESI-MS image acquisition software and inspected for polarimetric heterogeneity indicative of pathology. This polarimetry contrast is used to determine regions of interest for MS analysis. For example, our previous work has shown that regions of increased depolarization correspond to necrotic tissue^33^. Following polarimetry-targeted acquisition of MS images, the slice was subjected to H&E staining and microscopy evaluation. Importantly, DESI-MS uses a 1:1 mixture of acetonitrile and dimethylformamide as a solvent, which is compatible with histology needed for post-MS pathology evaluation (see below).

To assess the robustness of this workflow, and to illustrate the time-savings afforded by polarimetry guidance, we selected 8 regions of interest (6 ROIs of 1 mm^2^ and 2 ROIs of 4 mm^2^) from polarimetry-suggested necrotic, viable and border regions within the tissue slice, and directed our DESI-MS analysis to these regions. The results shown in Fig. 2 illustrate qualitative agreement between DESI-MS and depolarization results. Tissue areas that exhibit low depolarization, shown to correspond to viable cancer regions by H&E assessment, predominantly contain *m/z* 391.25 (viable cancer marker, green ROIs)^33^. Elevated depolarization regions, corresponding to necrotic cancer from H&E assessment, exhibit high levels of *m/z* 572.48 ([Cer(d34:1) + Cl]^−^, necrotic cancer marker, red ROIs)^33^. Further, polarimetrically-identified border regions revealed a mixture of viable and necrotic cancer markers as identified by MS. In terms of time savings, targeted DESI-MS analysis of these 8 polarimetry-selected ROIs took ~8 minutes total. For comparison and validation, the full-slide DESI-MS ion images for *m/z* 572.48 and *m/z* 391.25 are also presented in Fig. 2; these took ~6 hours to generate at 100 μm resolution. While faster MS data acquisition can be achieved using lower spatial resolution or sparser sampling of the entire slide surface, guided acquisition of high resolution data from key regions of interest such as the cancer border may provide a reasonable balance between data quality and experimental analysis time.

**Figure 3:**
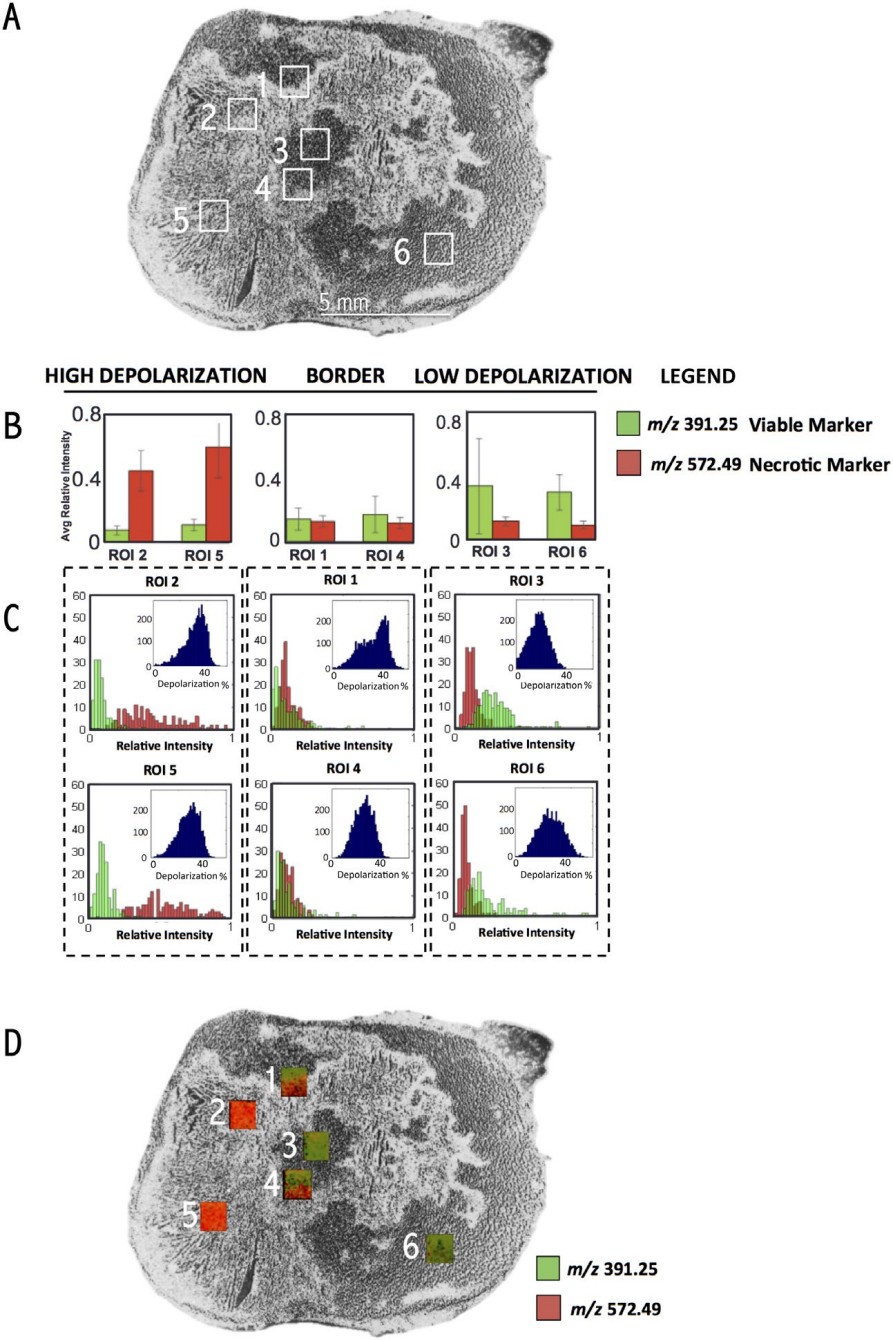
Quantitative assessment of concordance between polarimetric and DESI-MS signals. (**A**) Grayscale polarimetric heterogeneity image of the analyzed slice with the six small targeted ROIs highlighted. This image is the same polarimetric image shown in Fig. 2, reproduced here for better presentation of the assignment of ROIs 1–6 used in this quantitative assessment. (**B**) Average relative ion intensity of markers corresponding to necrotic (red, *m/z* 572.48) and viable (green, *m/z* 391.25) cancer tissue. ROIs are grouped based on the depolarization of the tissue analyzed. The viable cancers ROIs with lower depolarization values contain a greater abundance of viable cancer marker ion, and the opposite is true for necrotic centers (revealed by elevated depolarization) where the relative abundance of necrotic cancer marker ion is largest. Border regions show equal representation of both populations (**C**) Histograms of the pixel-wise distribution of relative ion intensity of each marker with inset histograms of the distribution of depolarization. The histogram distributions match that of average values shown in (**B**) both in MS and polarimetry, with correspondence between two techniques. (**D**) DESI-MS molecular images of *m/z* 391.25 (marker for viable cancer sites, in green) and *m/z* 572.48 (marker for necrosis cancer sites, in red) for ROIs selected in (**A**) overlaid on same polarimetric heterogeneity image in grayscale. MS images of each ROI overlaying the necrotic and viable markers. In areas with high depolarization the necrotic marker is significantly more intense while in regions with low depolarization the viable marker is more intense. In border regions, the intensities are roughly equivalent.

Quantitative concordance between polarimetry and guided DESI-MS imaging is examined in Fig. 3. The top panel shows an enlarged black and white (B&W) view of the Mueller matrix-derived depolarization image of the 10 μm slice from Fig. 2, exhibiting excellent tissue contrast. Superimposed on the image are six 1 mm^2^ ROIs, selected on the basis of polarimetric contrast, where polarimetry-guided DESI-MS imaging was performed. Tissue ROIs with low depolarization (ROIs 3 and 6, right column) exhibited a 3–4 fold greater average relative abundance of the viable cancer marker ion *m/z* 391.25 compared to the necrotic cancer marker ion *m/z* 572.48. Regions of high depolarization (ROIs 2 and 5, left column) contained a 5–6 fold higher average abundance of the necrotic cancer marker *m/z* 572.48 compared to the viable cancer marker *m/z* 391.25. Border regions (ROIs 1 and 4, middle column) contained mixed populations of both necrotic and viable cancer markers. The consistency between polarimetric contrast and detailed DESI-MS viable/necrotic signatures in these selected regions is encouraging, in the context of having the former (wide field and rapid) guide the latter (point-scanning and thus slower, but accurate).

A detailed pixel-wise histogram analysis of both MS ion abundances and polarimetry depolarization results is shown in Fig. 3C. ROIs 2 and 5, containing necrotic tissue, exhibited a wide distribution of medium to high relative ion abundances for the necrotic marker, peaking around 0.45, and a very tight (but less populous) distribution of viable cancer marker ion abundance peaking around 0.15. The corresponding depolarization histogram showed a preponderance of pixels with relatively elevated depolarizations, peaking at ~35%. As expected, viable cancer regions (ROIs 3 and 6) showed opposite trends: MS-measured ion abundance of the viable cancer marker peaked at around 0.35, and that of the necrotic cancer marker was lower, around 0.13. Polarimetry histograms reflected the lower depolarization for this region predominantly comprised of viable cancer tissue, peaking at ~20% (compared to 35% for necrotic regions). The transition border ROIs, 1 and 4, containing a mixed population of necrotic and viable cells, resulted in both *m/* *z* ion abundance distribution values peaking around 0.15. The depolarization histograms also exhibited intermediate values centered around 25–30% (notably exhibiting an approximately bimodal behavior for ROI 1). The concordance between ion abundance and depolarization histograms shown in Fig. 3C points to the consistency of the combined methodology for the assessment of tissue heterogeneity. Figure 3D partially reproduces the DESI-MS results shown in the workflow (Fig. 2D) and expands it for clarity of discussion. Here, polarimetry-MS concordance is easily visualized with overlays of the necrotic (red) and viable (green) MS markers in each ROI, and in the border regions the MS markers divide along the lines predicted by polarimetry. Additional experiments and histogram analyses will furnish probability distributions describing the relationship between polarimetry and MS, potentially yielding quantitative “likelihood of pathology” maps for polarimetric guidance of MS.

**Figure 4:**
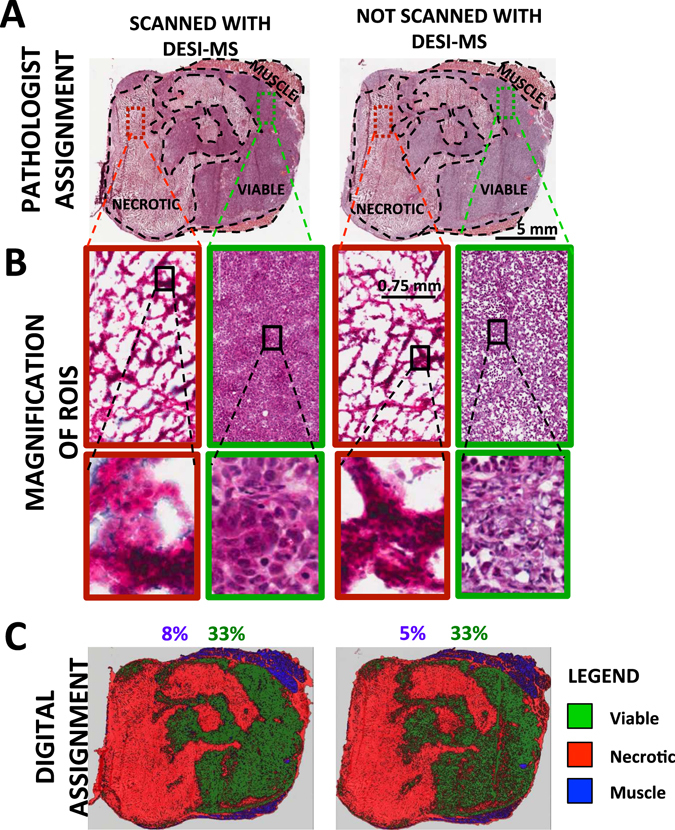
DESI-MS solvent spray has little impact on the suitability of post DESI-MS stained tissue slices for pathologic assessments. Two consecutive 10 μm slices of breast cancer tissue were obtained. The section to the left (section 1) was imaged with DESI-MS using a 1:1 mixture of acetonitrile:dimethylformamide (ACN:DMF) for lipid profiling in the negative ion mode. In contrast, the section to the right (section 2) was not exposed to DESI-MS solvent spray. (**A**) H&E images of the slices used in this assessment. (**B**) Zoomed in views of staining results. Necrotic regions appear in the red boxes, and viable regions appear in the green boxes. DESI-MS solvent treatment resulted in aberrant eosin staining of the viable cancer cells. (**C**) Training ROIs from section 2 (not scanned with DESI-MS) were used to perform automated segmentation of section 1 (which had come in contact with DESI-MS solvent spray prior to staining) with false colouring of the necrosis area (red), viable cancer area (green) and healthy tissue muscle (blue). The results of the morphometric analysis reveal that the relative surface area, and thus the gross morphology, of the viable cancer site (green) remain unchanged between DESI-MS imaged slice and the control.

A concern with the proposed workflow is that DESI-MS solvent spray may alter tissue morphology or biochemistry, making post-MS histological analysis on the same slide impossible. This has led to the development of histologically compatible solvent systems for minimally destructive DESI-MS imaging of tissues^28^. Here, to add more precision to these previous demonstrations^28^ and to address any potential criticism, two consecutive 10-μm-thick tissue slices were examined by a pathologist and analyzed by H&E based digital pathology. The morphometric methods employed in the latter allow an assessment of the potential alteration to tissue structure during DESI-MS in a quantitative manner. One slice was first subjected to DESI-MS and then subjected to histologic analysis, while the other did not come in contact with DESI-MS solvent and was used as a control. Figure 4A shows the resultant digitized H&E images, with regions of necrotic, viable and muscular tissue outlined (based on pathologist examination). Magnified images of the red and green-bordered rectangles are shown in 4B. Despite slightly darker staining of intercellular regions with eosin in the tissue slice that had been exposed to DESI-MS solvent, the pathologist was easily able to distinguish necrotic from viable tissue. Detailed morphometric analysis using classifiers derived from a control slide yielded similar results for delineating necrotic and viable cancer regions (Fig. 4C). A minor 3% increase in predicted total surface area of the muscle tissue was seen, possibly due to artifacts caused by minor tissue folding at the edges of the sections, and further contributed to by overall darker appearance of the eosin stain (possibly due to DESI-MS). Nevertheless, we conclude that histology assessment post MS, using traditional human-observer and detailed digital methods, is hardly affected. In summary, the proposed workflow for the analysis of thin tissue slices combines individual advancements detailed above to improve sensitivity and increase cross-platform compatibility in sample preparation requirements can be implemented on a single tissue slice. This sets our current work apart from our previous proof-of-principle demonstration that required more than one slice to meet differential sensitivity and preparation requirements for polarimetric imaging, DESI-MS imaging and histopathology. Performing guidance and MS imaging on the same tissue slice is a significant advance due to the error in margins that can result from consecutive slices. Furthermore, polarimetric measurements in the reflection mode, not reported in our previous work, expand the utility of guided MS sampling by wide-field imaging to thick, *in situ* tissue samples.

It is worth noting that the polarimetry trend could be potentially confounding given our other findings which demonstrate that healthy tissue also shows increased depolarization compared to cancerous tissue^18^. That is, necrotic tumour tissue (which shows elevated depolarization relative to viable tumour tissue) could potentially be mistaken as healthy tissue if these tissue types demonstrate similar depolarizations. To avoid this confusion, the relative scale of the depolarization trends must be such that healthy tissue is more depolarizing than necrotic tissue, which in turn is more depolarizing than viable tumour. In fact, this trend is suggested by the region of healthy muscle tissue in the upper right of Fig. 2B, which shows significantly higher depolarization than both the viable and necrotic tumour regions. A more detailed analysis with all three of these tissue states (healthy, viable tumour and necrotic tumour) present in the same tissue type (i.e, healthy lymph node present instead of healthy muscle) is needed to firmly establish the relative scale of depolarization trends.

Having demonstrated the viability of polarimetry-guided MS for thin-tissue slide scanning, we now investigate thick-tissue examinations relevant to whole (bulk) *ex vivo* specimen profiling and future *in-vivo* intraoperative applications. DESI-MS analysis cannot be easily performed on thick or uneven tissue. Therefore, we developed a hand-held laser ablation device based on Picosecond InfraRed Laser (PIRL) technology that we’ve demonstrated to be a suitable MS desorption source when coupled to a post ionization method^31^. PIRL ablation, shown to provide rapid extraction of molecules from tissue^45^, including molecules already in solvated ionic state such as phospholipids and fatty acids, was coupled to a home-made soft thermal ionization interface capable of desolvating ionized tissue materials. A flexible Tygon tube extends the collection capillary of a modified commercial DESI-MS interface, and is heated to provide desolvation and evaporative thermally induced ionization (Figure S1). As shown in Figure S2, as few as 5–10 s of point sampling over an area of ~2 mm^2^ with PIRL ablation is sufficient to correctly classify phospholipid and fatty acid profiles of healthy mouse organ tissues. Figures S3–S7 show the reproducibility of mouse organ PIRL-MS profiles obtained from 4 independent mice, with some repetitions therein. We were able to detect tissue specific *m/z* values (allowing tissue classification) in all independent repetitions. The plume transport and ionization for PIRL MS analysis herein is completed without a Rapid Evaporative Ionization Mass Spectrometry (REIMS) interface used for real time analysis of electrocautery plume or other surgical aerosols including ablation plume for other laser systems^9, 10, 13, 14^. We, however, anticipate integration with REIMS interface is likely to increase the robustness of signal and reproducibility of plume collection due to increased suction by Venturi action, and further would allow infusion of matrix solvent to optimize desolvation/ionization.

To investigate whether PIRL-MS spectra had statistical relevance for discriminating between tissue types we subjected the PIRL-MS spectra of various mouse tissues from 4 independent mice to Partial Least Squares Discriminant Analysis (PLS-DA). Figure S8 shows the PLS-DA scores plot, successfully grouping tissue data points based on their PIRL-MS spectra (collected in 10 s). Real time MS profiling with PIRL ablation can thus be used to identify *in situ* tissue types in 10 s of sampling using the configuration shown in Figure S1. The success of PIRL-MS in rapid tissue profiling is largely due to efficient coupling of vibrational excitation of water molecules to ablative modes using impulsive deposition of heat through picosecond IR radiation^46^. The high efficiency in converting incident optical energy to ablation produces highly desolvated gas phase phospholipids and fatty acids. This vapour is readily ionizable upon slight desolvation with soft techniques such as thermal ionization or evaporative ionization (Figure S1).

**Figure 5:**
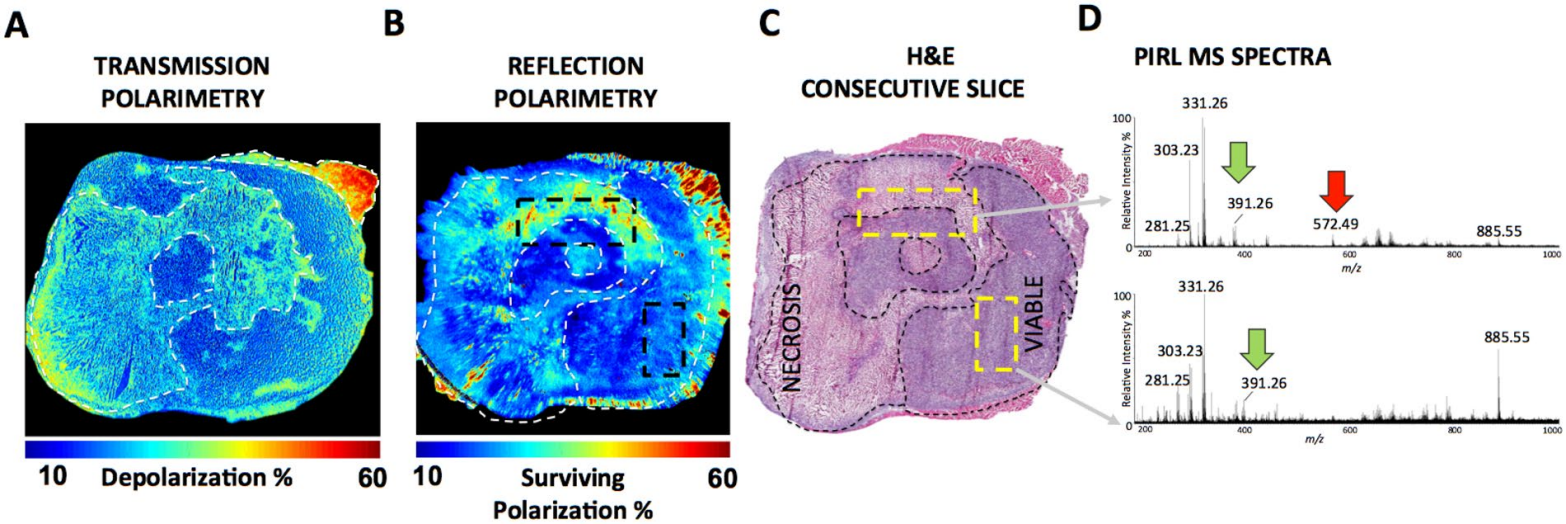
Guidance of PIRL-MS to areas of heterogeneity using polarimetric imaging of an *ex vivo* tissue slice in the reflection mode. The hand held laser MS sampling probe described in Figure S1 is visually guided to areas of polarimetric heterogeneity indicative of necrosis and viable cancer on a 50 μm tissue slice. (**A**) Same transmission polarimetry image shown in Fig. 2 from 10 μm tissue slice illustrating good contrast between necrotic and viable cancer sites within the tissue. (**B**) Polarimetric imaging of a 50 μm tissue slice in the reflection mode. This slice is a few hundred microns apart from the slice used in (**A**). (**C**) H&E image of a 10 μm slice consecutive to the 50 μm section subjected to polarimetric assessment in (**B**). (**D**) PIRL-MS analysis of 50 μm tissue slice based on reflection mode polarimetric imaging feedback. The laser ablation sites are highlighted on the H&E image given in panel (**C**). Following 5–10 seconds of ablation of the tissue and real time analysis of the ablation plume, MS spectra are generated. The viable cancer maker ion of *m/z* 391.25^33^ is present in areas of low depolarization (for transmission) and low surviving polarization (for reflection). It is expected that regions displaying low depolarization in transmission will appear as regions of low surviving polarization in reflection geometry (see text). We thus show an image of the “surviving polarization” for the reflection image so that contrast similar to the transmission image is easily visualized. The laser ablated region that contained both necrotic and viable cancer from polarimetry feedback resulted in the detection of both necrotic (*m/z* 572.48) and viable (*m/z* 391.25) biomarker ions^33^. The necrotic (red) and viable (green) regions are highlighted in transparent overlays. In addition, all relevant biomarker ions for breast tissue *m/z* 281.25 [FA(18:1)-H]^−^ (oleic acid), 303.23 [FA(20:4)-H]^−^ (arachidonic acid)^3, 7, 50^, *m/z* 331.26 [FA(22:4)-H]^−^ (adrenic acid)^3, 7, 50^ are detected with PIRL-MS in 10 s of sampling breast cancer tissue.

With a hand held MS point sampling device suitable for molecular profiling of thick tissue, we now investigate polarimetry of thick tissue for possible *in situ* or *in vivo* MS guidance. Figure 5A reproduces the transmission polarimetry image obtained from the thin 10 µm slice shown in Fig. 2, for side-by-side comparison with the reflection mode image from a 50-µm-thick slice shown in Fig. 5B. While these slices are hundreds of microns apart and of significantly different thicknesses, relative contrast between viable and necrotic cancer sites seen with the two approaches is overall similar. Note that in transmission geometry, low depolarization is observed where most light passes through the sample without much scattering and hence polarization is preserved. However, in reflection mode from thick tissues, light backscattered from such regions is highly depolarized (i.e., surviving polarization is low). We have previously observed this depolarization inversion dependence on the detection geometry in relatively low scattering regimes; for moderate to high scattering regions, light exiting the sample at any angle undergoes a similar number of scattering events, and thus depolarization shows far less detection geometry dependence^47^. To acknowledge that regions of low depolarization in thin-sample transmission become regions of low surviving polarization in thick-sample reflection, we display “surviving polarization” in Fig. 5B. Thus appropriate choice of experimental guidance metrics readily illustrates the similarity in polarimetric contrast offered by both reflection and transmission polarimetry. Thick-tissue reflection results show lower resolution and contrast between viable and necrotic regions, but these may still be sufficient for MS guidance. H&E histology of an adjacent 10 µm slice (5C) shows that the reflection polarimetry from thick tissue (5B) provides a reasonable estimate of viable vs. necrotic cancer differentiation, based on differences in the polarization properties. Decreases in resolution and contrast in reflection mode imaging are expected, arising from the confounding effects of underlying tissue on the polarimetric signals, and from the previously mentioned issue of spatial (depth) heterogeneity of tissue. Some of these confounders can be accounted for (as will be pursued in future work) via our effective sampling depth and path-length estimation from polarization-sensitive Monte Carlo modeling^48^ and via axial heterogeneity detection and corrections^49^. So while additional research will likely improve the contrast and resolution of reflection-mode polarimetric images, this initial result still suggests suitability of the approach for MS guidance in a thick-tissue setting.

We now couple our point-sampling PIRL-MS technology with reflection polarimetry, attempting to identify areas of necrosis in metastatic breast tumours and verify the presence of necrotic cancer marker ion *m/z* 572.48. To first order, we hypothesize that the surface of the 50 µm tissue slice used herein can be likened to a flat tissue surface exposed through cutting with a scalpel. The surface of this tissue slice was imaged with reflection polarimetry (Fig. 5B) and then subjected to subsequent laser ablation and real time sampling with MS, using polarimetric guidance. PIRL-MS sampling of the region with low surviving polarization, as seen in reflection polarimetry (Fig. 5C, far right ROI), yielded MS spectra consistent with that of viable tissue, illustrating significant presence of viable m/z 391.26 ion and absence of the m/z 572.48 necrosis marker (Fig. 5D, bottom)^33^. In the region of mixed polarization heterogeneity (Fig. 5C, top ROI), PIRL-MS results show the presence of both viable and necrosis markers (Fig. 5D, top). This consistency between reflection polarimetry and PIRL-MS, analogous to transmission polarimetry and DESI-MS results in thin tissues, is encouraging and suggests that both hybrid implementations are potentially valid. Of special note is the presence of many common fatty acid and phospholipids in PIRL-MS spectra of breast cancer reported previously using other MS technologies^3, 7, 18, 50^. This similarity further speaks to PIRL's ability to extract ionized molecules from the tissue. Namely, all the known breast cancer biomarker ions of *m/z* 281.25 [FA(18:1)-H]^−^ (oleic acid), 303.23 [FA(20:4)-H]^−^ (arachidonic acid)^3, 7, 50^, and *m/z* 331.26 [FA(22:4)-H]^−^ (adrenic acid)^3, 7, 50^ were detected with PIRL-MS.

Figure S9 summarizes the two polarimetry-guided MS implementations described in this study for both thin tissue sections (Figure S9A) using DESI-MS and polarimetric imaging in transmission mode, and thick tissue (Figure S9B) using PIRL MS and polarimetric imaging in the reflection mode. The significant improvement reported here, relative to our previous proof-of-principle demonstrations using thin tissue sections^18^, is that the entire work flow is now possible with a single tissue slice. This is enabled by the described MS and polarimetry technology improvements and the utilization of a DESI-MS solvent mix compatible with histology staining. Future work includes quantification of sensitivity and specificity of polarimetric guidance in a variety of tissue pathologies, and engineering of the two technologies into a single integrated system.

The reflection polarimetry and PIRL-MS point sampling of polarimetry-targeted sites shown in Fig. 5 is particularly suitable for examining thick tissues *ex-vivo* (e.g., entire surface of whole breast lumpectomy specimen, intraoperatively during breast-conserving surgery) and for potential *in-vivo* deployment. This approach, summarized in Figure S9B, is less developed and its feasibility is illustrated here with initial results on thick flat tissue. Near-term work plans include improvements in polarimetry contrast and resolution, image processing for PIRL coordinate co-registration and probe guidance, and further validations with histology in various tissues. Longer-term research includes assessment of performance *in vivo* and addressing the confounding effects, such as surface roughness, blood, tissue motion and presence of layered heterogeneous tissues.

To summarize, we have demonstrated the feasibility of polarimetry-guided MS for assessment of biological tissues in two different arrangements of clinical relevance, with the over-arching goal of obtaining detailed compositional maps of tissues in a robust and time-efficient manner. Upon further optimization of enabling technologies and work-flow process logistics, these developments may have a significant impact in surgical oncology and other clinical settings. Provided sufficient polarimetric contrast exists between disease and healthy tissue, we anticipate that our platform will be capable of rapidly characterizing a variety of disease states.

## Electronic supplementary material

Supplementary Information
