# Supplementary material for: Optimized Mass Spectrometry Analysis Workflow with Polarimetric Guidance for ex vivo and in situ Sampling of Biological Tissues
Source: Sci Rep. 2017 Mar 28;7:468. doi: 10.1038/s41598-017-00272-y (PMC5428042; doi:10.1038/s41598-017-00272-y)
Supplement: Supplementary file 1 — Supplementary Information [file 41598_2017_272_MOESM1_ESM.pdf]

Supplementary Information

Optimized Mass Spectrometry Analysis Workflow with Polarimetric Guidance for *ex vivo* and *in situ* Sampling of Biological Tissues

Michael Woolman[a], Adam Gribble[b], Emma Bluemke[a], Jing Zou[a], Manuela Ventura[a], Nicholas Bernards[a], Megan Wu[c], Howard J. Ginsberg[a,d,e], Sunit Das[c,d,e], Alex Vitkin[c,f,g] and Arash Zarrine-Afsar [a,b,d,e,*]

[a]Techna Institute for the Advancement of Technology for Health, University Health Network, Toronto, ON, M5G-1P5, Canada
[b]Department of Medical Biophysics, University of Toronto,101 College Street Suite 15-701, Toronto, ON, M5G 1L7, Canada
[c]Peter Gilgan Centre for Research and Learning, Hospital for Sick Children, 686 Bay Street, Toronto, ON, M5G-0A4 , Canada
[d]Department of Surgery, University of Toronto, 149 College Street, Toronto, ON, M5T-1P5, Canada
[e]Keenan Research Center for Biomedical Science, Li Ka Shing Knowledge Institute, St. Michael's Hospital, 30 Bond Street, Toronto, ON, M5B-1W8, Canada
[f]Department of Radiation Oncology, University of Toronto, 610 University Avenue, Toronto, Ontario M5G 2M9, Canada
[g]Division of Biophysics and Bioimaging, Ontario Cancer Institute, University Health Network, 610 University Ave, Toronto, ON M5G 2M9
[i]Institute of Biomaterials and Biomedical Engineering, University of Toronto, 164 College Street, Toronto, ON M5S 3G9
*Corresponding author: arash.zarrine.afsar@utoronto.ca

Table of Content

Figure S1 .... **S3**
Figure S2 .... **S4**
Figure S3 .... **S5**
Figure S4 .... **S6**
Figure S5 .... **S7**
Figure S6 .... **S8**
Figure S7 .... **S9**
Figure S8 .... **S10**
Figure S9 .... **S11**
References **S12**

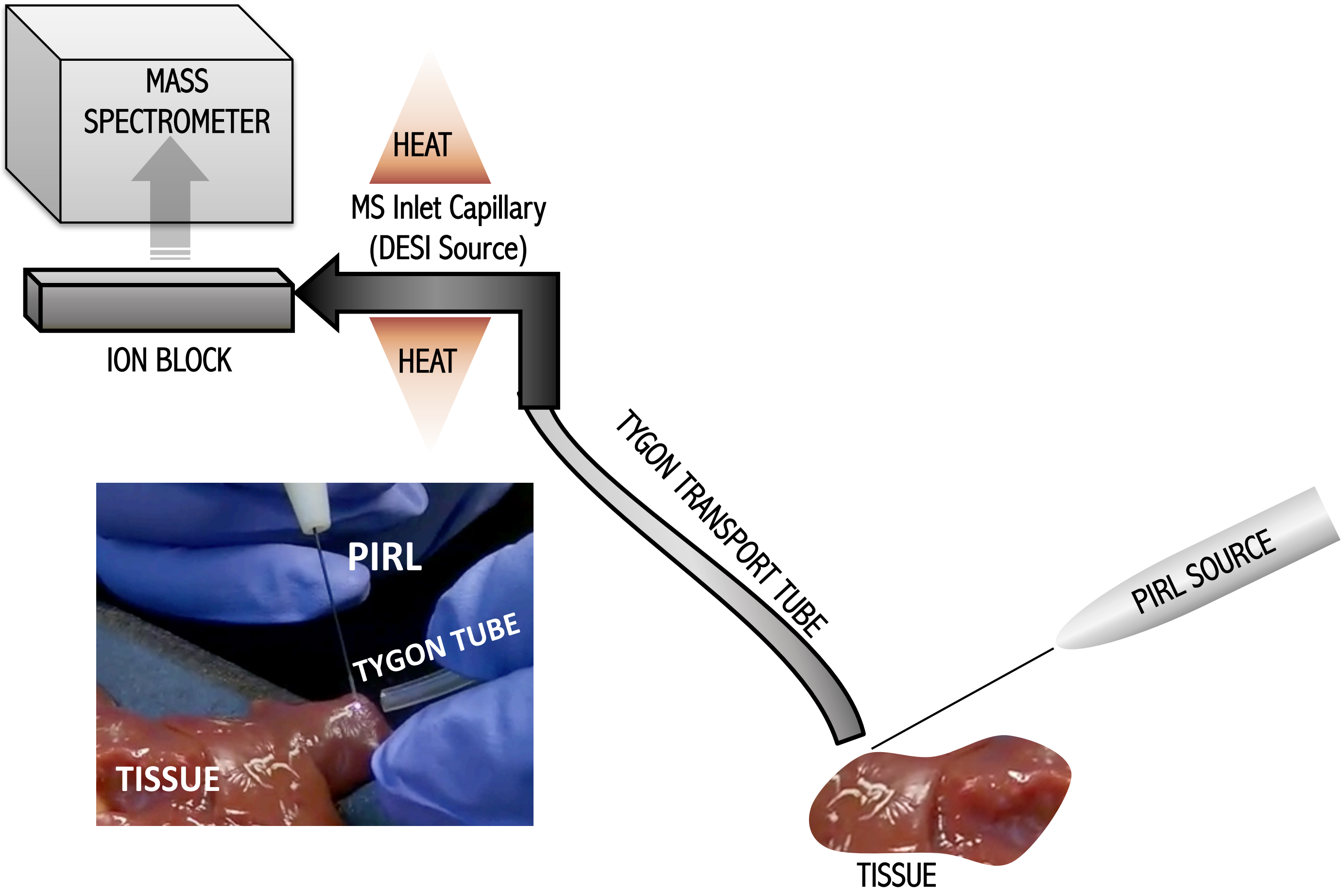

Figure S1. Schematics of SMART laser probe based on Picosecond InfraRed Laser (PIRL) ablation soft ionization mass spectrometry (MS). The MS inlet collection capillary of a commercial DESI-MS interface is extended with a 2 m long Tygon tube and heated to provide desovlation and soft ionization of ablated material before direct injection into the ion block. This allows near real time acquisition of MS spectra from ~ 0.05 mm^3 of tissue material removed.

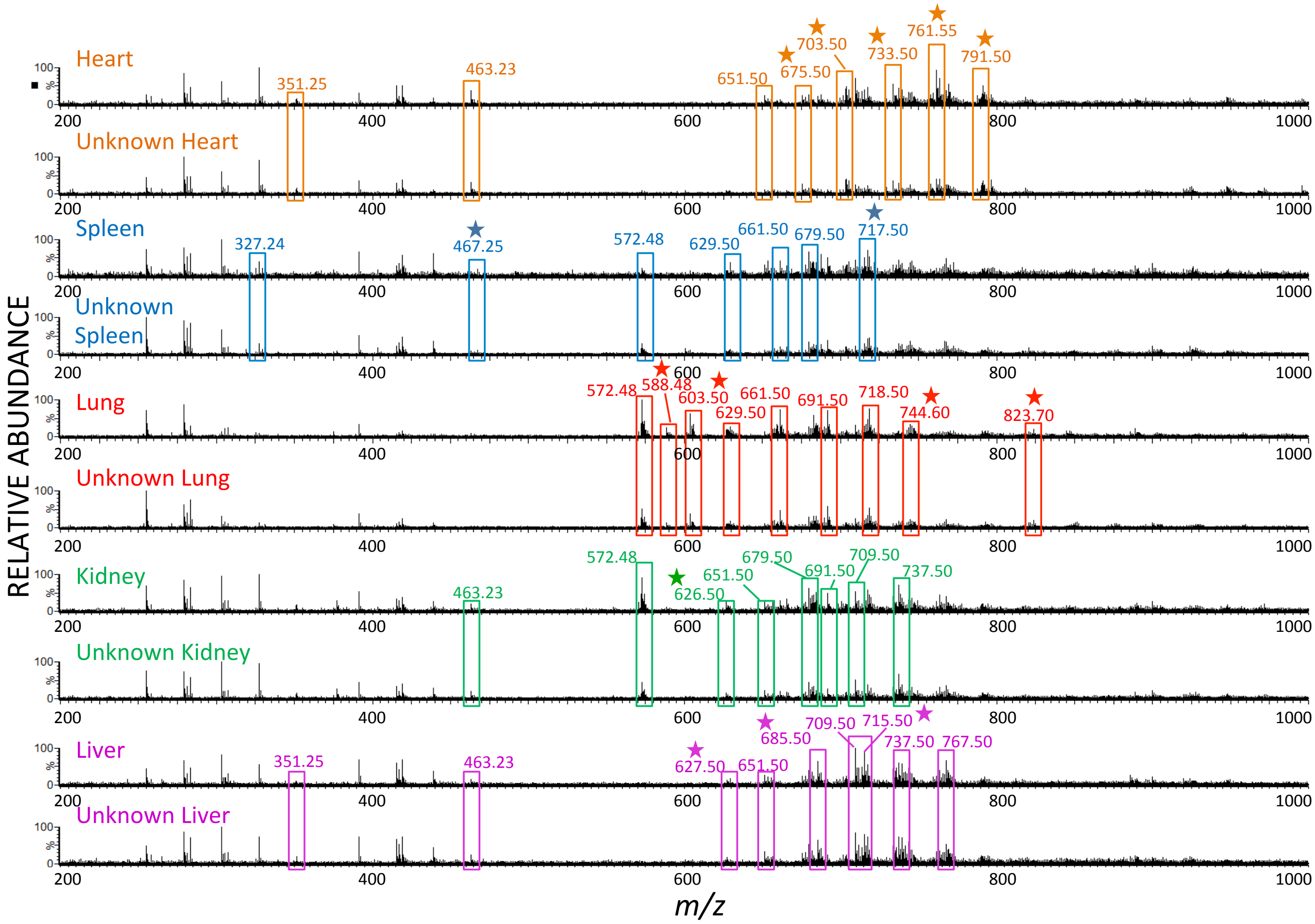

Figure S2. Identification of mouse organs with ~10 seconds of sampling with hand held PIRL ablation MS sampling device. The Mass Spectrometry (MS) lipid profiles for a variety of mouse organs collected in only 10s of sampling with picosecond infrared laser ablation, soft ionization mass spectrometry is presented along with the mass to charge (*m/z*) values (highlighted) that characterize each organ. The coincidence between these *m/z* values and those from known organs is used to classify organ types in blind experiment. Figures S3-S7 show how reproducible tissue spectra were using organs from independent mice. In each panel, the *m/z* value(s) unique to each organ type are highlighted with a star.

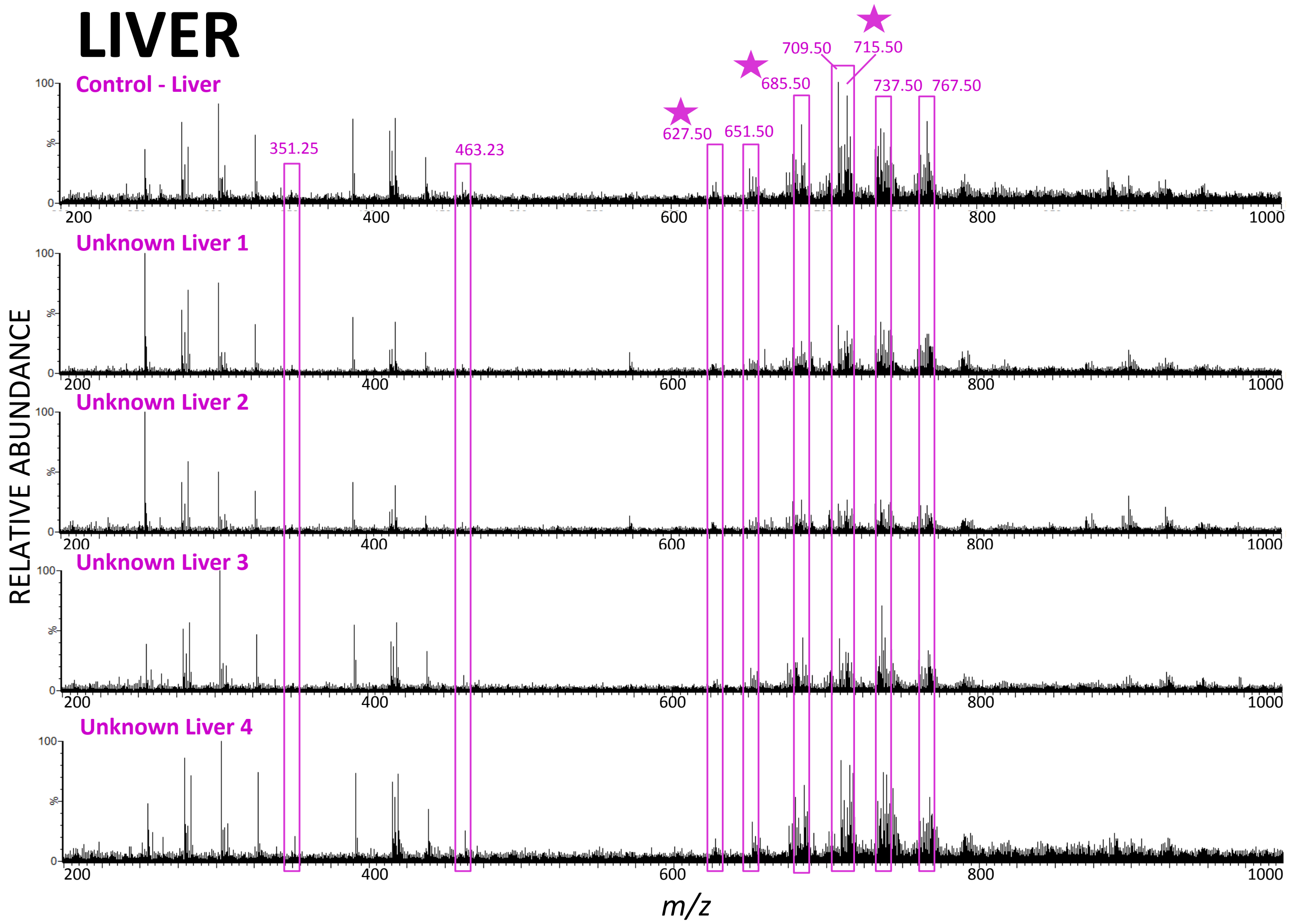

Figure S3. PIRL soft ionization MS analysis of mouse liver with ~10 seconds of *in situ* sampling. The Mass Spectrometry (MS) lipid profile collected in 10s of sampling with picosecond infrared laser ablation, soft ionization mass spectrometry is presented along with unique mass to charge (*m/z*) values (highlighted) that characterize this tissue. The *m/z* value(s) unique to liver are highlighted with a star.

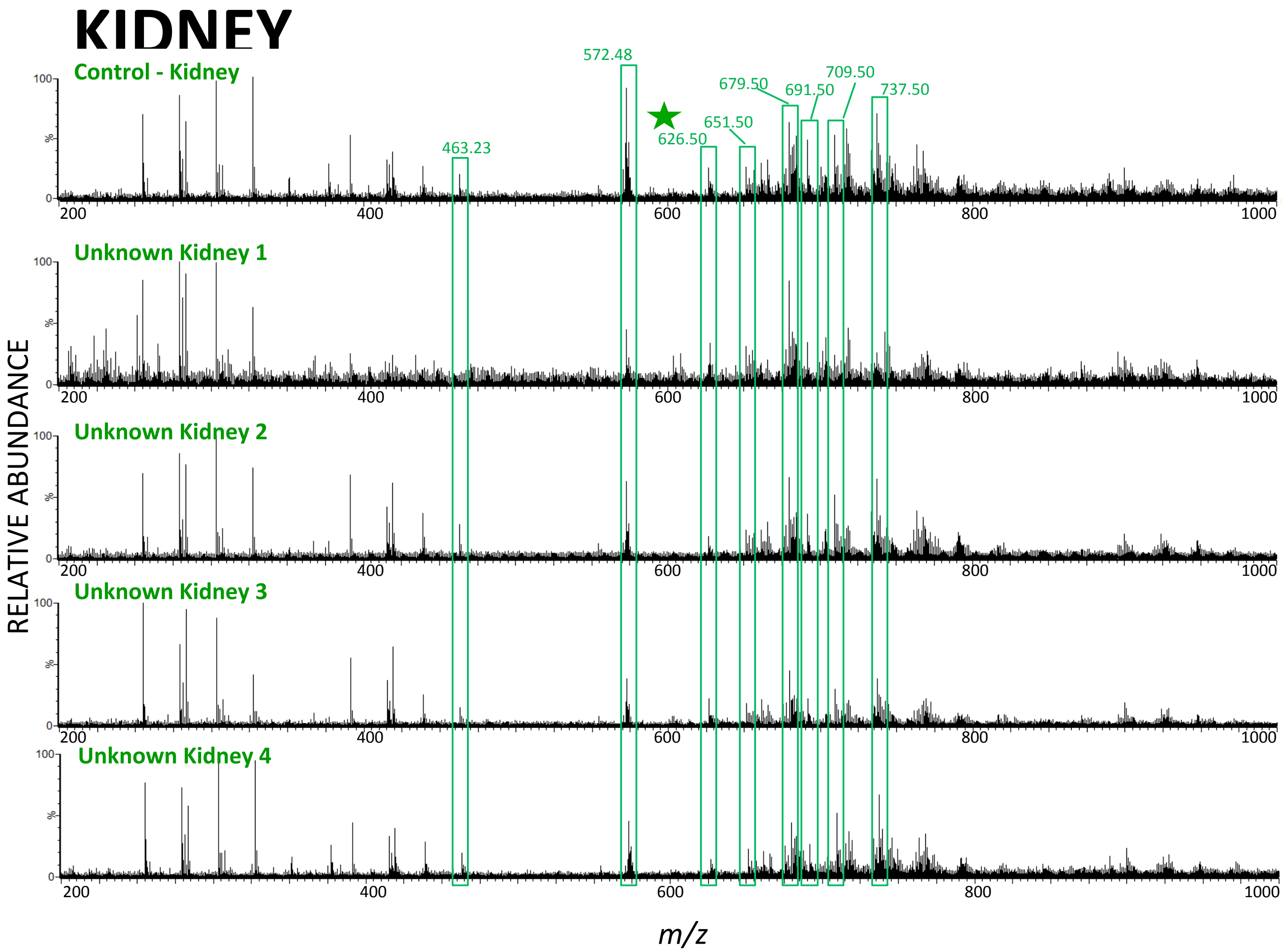

Figure S4. PIRL soft ionization MS analysis of mouse kidney with ~10 seconds of *in situ* sampling. The Mass Spectrometry (MS) lipid profile collected in 10s of sampling with picosecond infrared laser ablation, soft ionization mass spectrometry is presented along with unique mass to charge (*m/z*) values (highlighted) that characterize this tissue. The *m/z* value(s) unique to kidney are highlighted with a star.

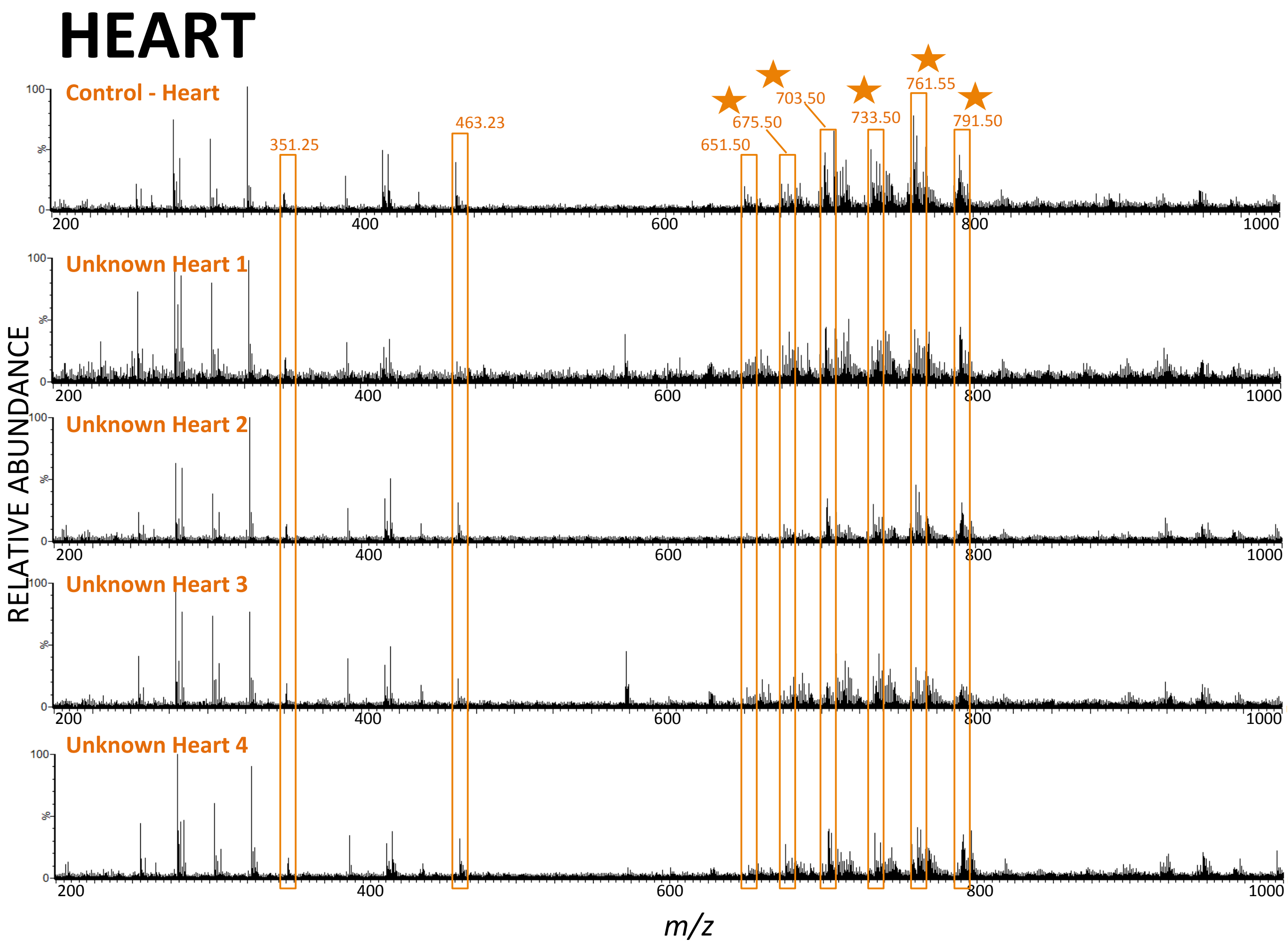

Figure S5. PIRL soft ionization MS analysis of mouse heart with ~10 seconds of *in situ* sampling. The Mass Spectrometry (MS) lipid profile collected in 10s of sampling with picosecond infrared laser ablation, soft ionization mass spectrometry is presented along with unique mass to charge (*m/z*) values (highlighted) that characterize this tissue. The *m/z* value(s) unique to heart are highlighted with a star.

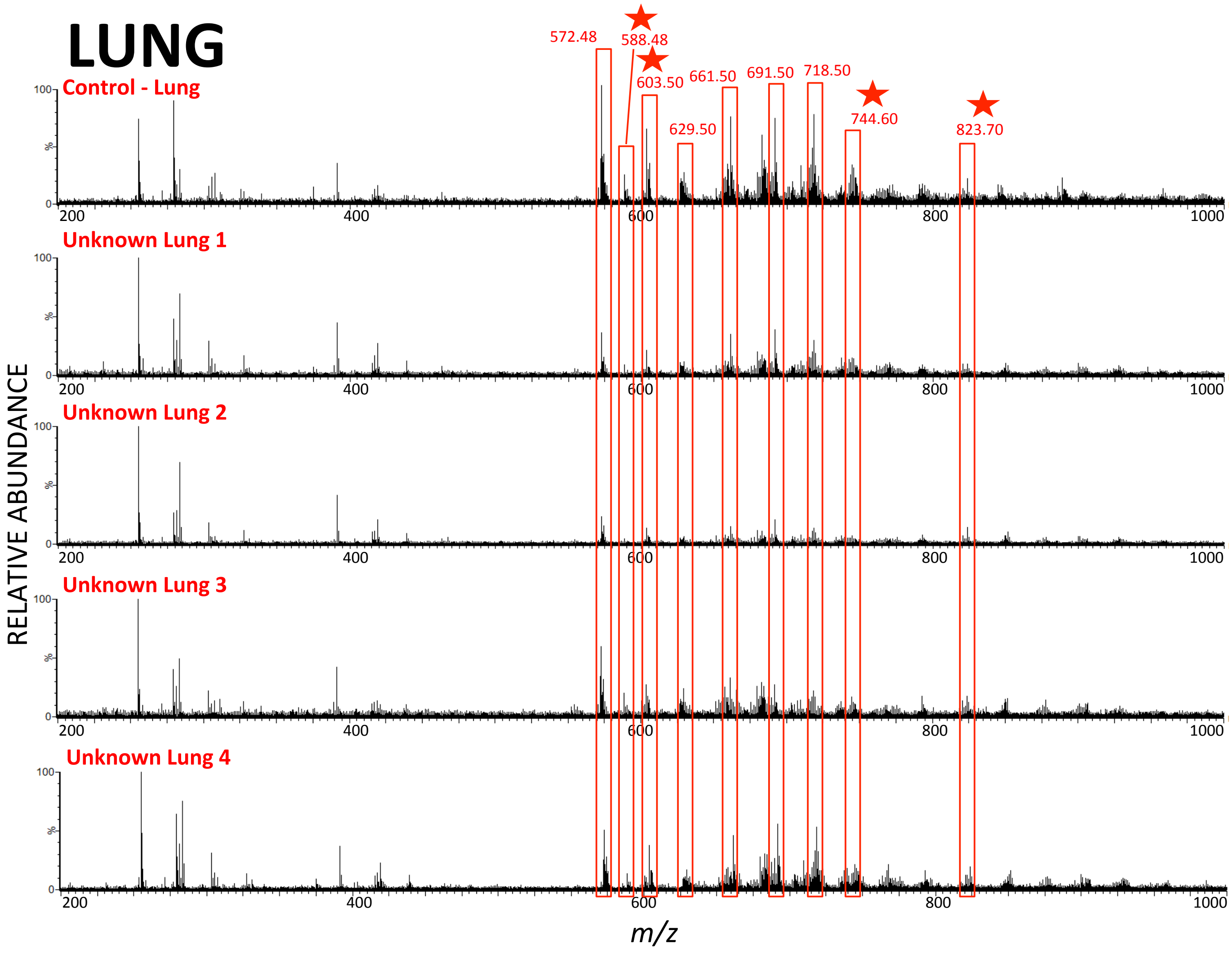

Figure S6. PIRL soft ionization MS analysis of mouse lung with ~10 seconds of *in situ* sampling. The Mass Spectrometry (MS) lipid profile collected in 10s of sampling with picosecond infrared laser ablation, soft ionization mass spectrometry is presented along with unique mass to charge (m/z) values (highlighted) that characterize this tissue. The m/z value(s) unique to lung are highlighted with a star.

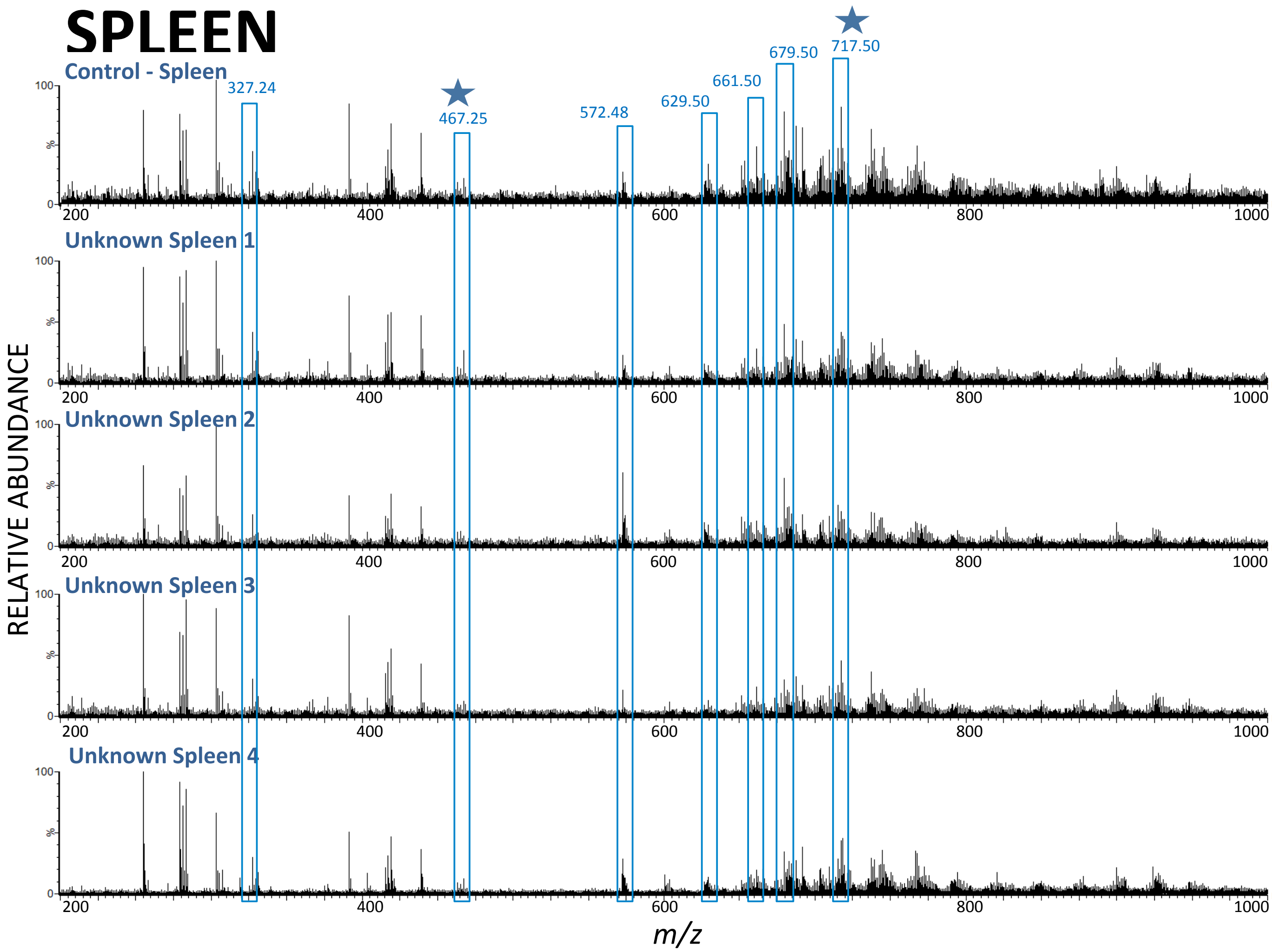

Figure S7. PIRL soft ionization MS analysis of mouse spleen with ~10 seconds of *in situ* sampling. The Mass Spectrometry (MS) lipid profile collected in 10s of sampling with picosecond infrared laser ablation, soft ionization mass spectrometry is presented along with unique mass to charge (*m/z*) values (highlighted) that characterize this tissue. The *m/z* value(s) unique to spleen are highlighted with a star.

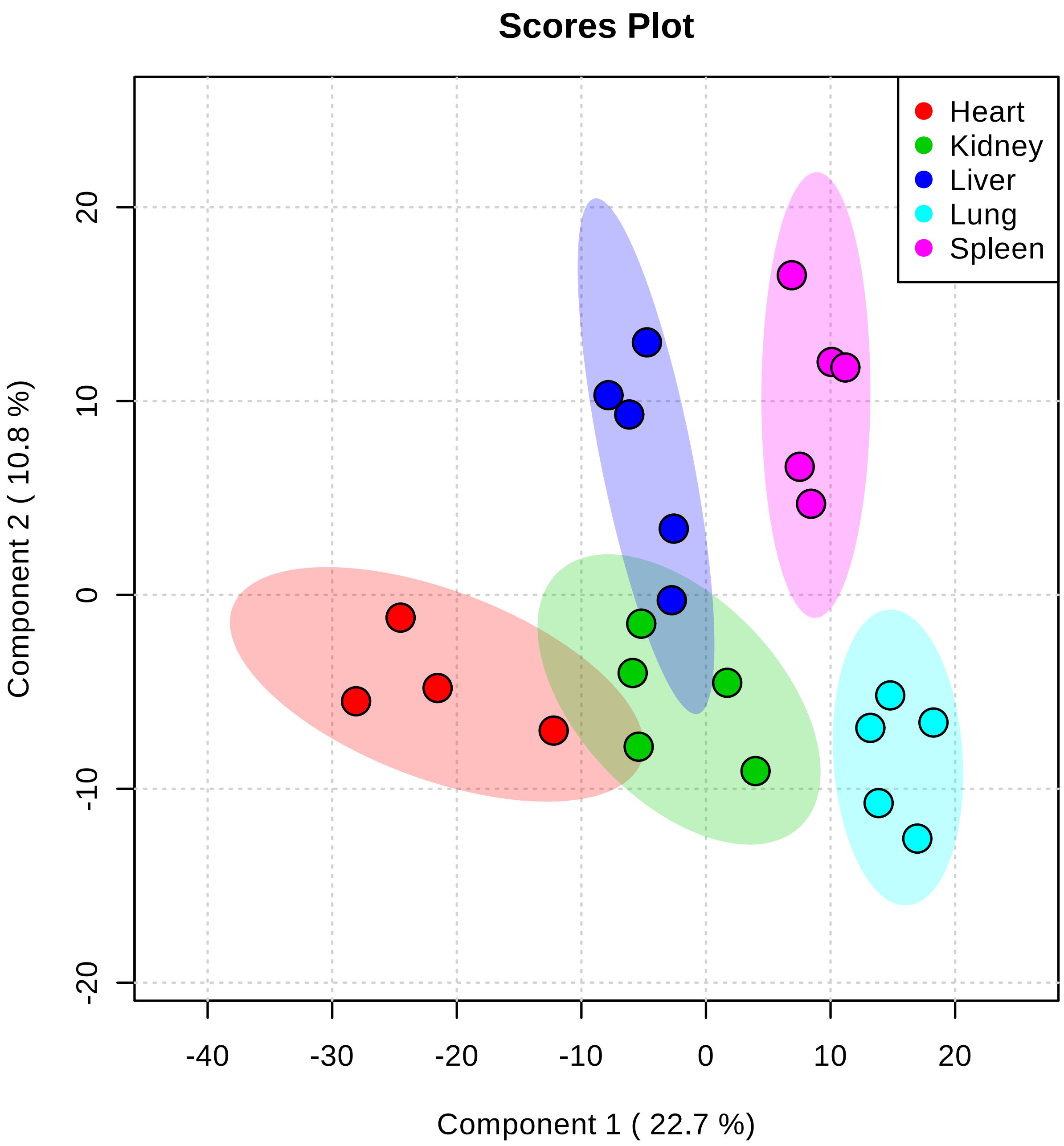

Figure S8. Statistical discrimination between PIRL MS profiles of different mouse tissues examined in this study. The Mass Spectrometry (MS) lipid profiles collected in 10s of sampling with picosecond infrared laser ablation, soft ionization mass spectrometry was subjected to Partial Least Squares Data Analysis (PLS-DA) method using the MetaboAnalyst platform. The scores plot with 96% confidence interval is shown that suggests clear grouping between data for each organ from 4 independent mice.

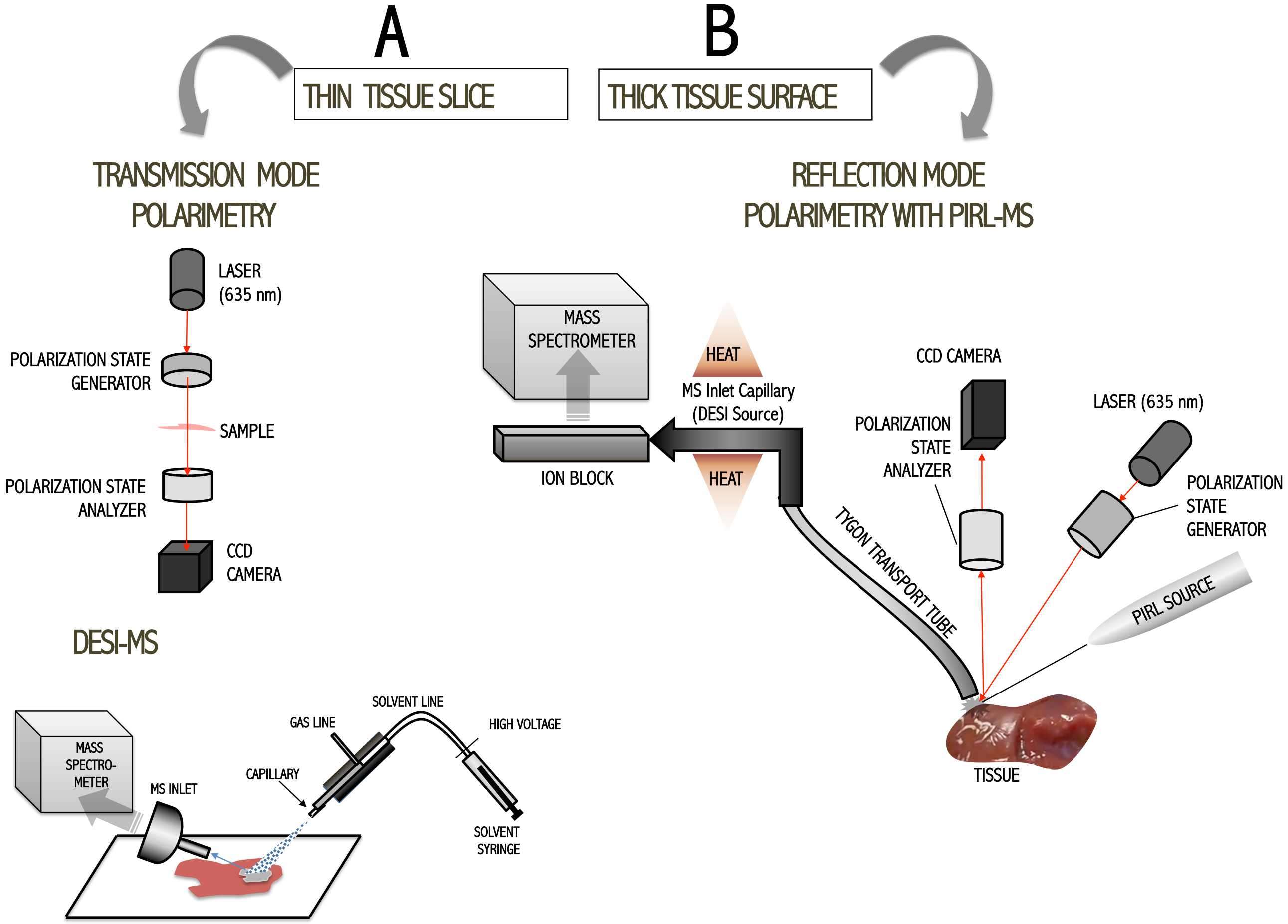

Figure S9. Workflows for polarimetry guided MS sampling of *ex vivo* and *in situ* biological tissues. (A) The schematic setup for transmission polarimetry and DESI-MS are shown, including details of DESI-MS setup and polarimetric imaging. *Ex vivo* thin tissue slices will be subjected to transmission polarimetric imaging to reveal areas of polarimetric heterogeneity. The workflow proposed in Figure 2 will then be followed to guide targeted acquisition of MS images. (B) Schematics of the desired *in situ* setup are shown. *In situ* tissue will be subjected to reflection polarimetry imaging to reveal areas of polarimetric heterogeneity. These regions will then be directly sampled by PIRL-MS (details in figure S1) to collect molecular information. For completeness, we reproduce the schematics of PIRL-MS coupling also shown in Figure S1. Areas of polarimetric heterogeneity are shown in gray, to which the MS sampling probe is accordingly guided. The choice of polarimetry for this visualization is based on attributes such as sufficient contrast, being nondestructive to the tissue and providing a rapid wide-field overview of the tissue area to reveal areas of pathology.

References:

1 Calligaris, D. *et al.* Application of desorption electrospray ionization mass spectrometry imaging in breast cancer margin analysis. *Proc Natl Acad Sci U S A* **111**, 15184-15189 (2014).

2 Dill, A. L., Ifa, D. R., Manicke, N. E., Ouyang, Z. & Cooks, R. G. Mass spectrometric imaging of lipids using desorption electrospray ionization. *J Chromatogr B Analyt Technol Biomed Life Sci* **877**, 2883-2889 (2009).

3 Guenther, S. *et al.* Spatially resolved metabolic phenotyping of breast cancer by desorption electrospray ionization mass spectrometry. *Cancer Res* **75**, 1828-1837 (2015).
